# Blood Vessel Segmentation of Retinal Image Based on Dense-U-Net Network

**DOI:** 10.3390/mi12121478

**Published:** 2021-11-29

**Authors:** Zhenwei Li, Mengli Jia, Xiaoli Yang, Mengying Xu

**Affiliations:** School of Medical Technology and Engineering, Henan University of Science and Technology, Luoyang 471023, China; 190319221052@stu.haust.edu.cn (M.J.); yangxiaoli39@163.com (X.Y.); 200320221486@stu.haust.edu.cn (M.X.)

**Keywords:** U-Net, dense block, retinal image, blood vessels segmentation

## Abstract

The accurate segmentation of retinal blood vessels in fundus is of great practical significance to help doctors diagnose fundus diseases. Aiming to solve the problems of serious segmentation errors and low accuracy in traditional retinal segmentation, a scheme based on the combination of U-Net and Dense-Net was proposed. Firstly, the vascular feature information was enhanced by fusion limited contrast histogram equalization, median filtering, data normalization and multi-scale morphological transformation, and the artifact was corrected by adaptive gamma correction. Secondly, the randomly extracted image blocks are used as training data to increase the data and improve the generalization ability. Thirdly, stochastic gradient descent was used to optimize the Dice loss function to improve the segmentation accuracy. Finally, the Dense-U-net model was used for segmentation. The specificity, accuracy, sensitivity and AUC of this algorithm are 0.9896, 0.9698, 0.7931, 0.8946 and 0.9738, respectively. The proposed method improves the segmentation accuracy of vessels and the segmentation of small vessels.

## 1. Introduction

The retinal vascular system provides rich information about the state of the eye and is the only non-invasive imaging method to obtain visible blood vessels from the human body. Retinal vascular segmentation is of great significance for the diagnosis of fundus diseases [1]. As a result, retinal images have been widely used to detect early signs of systemic vascular disease. In order to facilitate the diagnosis of systemic vascular diseases, vessels need to be accurately segmented. Therefore, the automatic segmentation of retinal blood vessels from fundus images has become a popular research topic in the medical imaging field.

The existing traditional algorithms mainly implement retinal image vascular segmentation using the matched filter method [2], morphology method [3], and vascular tracking method [4]. Jaspreet et al. [5] proposed a segmentation method based on the Gabor filter. Wang et al. [6] proposed a novel divide-and-conquer funnel-structured classification framework for retinal vessel segmentation. To address the imbalance problem, Yan et al. [7] explore to segment thick vessels and thin vessels separately by proposing a three-stage deep learning model. FRAZ et al. [8] proposed a unique combination of vessel centerline detection and morphometric bit-plane section techniques to extract blood vessels from retinal images. SOARES et al. [9] proposed a method for automated segmentation of the vasculature in retinal images. The method produces segmentations by classifying each image pixel as vessel or non-vessel. SAFFARZADEH et al. [10] proposed a new method of retinal image vascular segmentation based on multi-scale line operator and K-means clustering. GU et al. [11] proposed a context encoder network to capture high-level information and preserve spatial information for 2D medical image segmentation. Choy et al. [12] proposed a new image segmentation algorithm based on an unsupervised fuzzy model. Zhang et al. [13] used Bayesian theory and multi-scale linear detection to activate, track and segment blood vessels. Orlando et al. [14] proposed a special model for vascular segmentation with a sensitivity of 0.7897 and 0.7277 on DRIVE and CHASE_DB1 datasets, respectively. However, traditional retinal vascular segmentation algorithms cannot accurately segment the images with different gray-scale characteristics. In recent years, deep learning has shown significant advantages in medical image analysis. A large number of deep learning-based vascular segmentation methods have also been proposed [15]. Wang et al. [16] proposed a supervised approach based on feature and ensemble learning. LISKOWSKI et al. [17] proposed a supervised segmentation technique that uses a deep neural network, trained on a large sample of examples preprocessed with global contrast normalization, zero-phase whitening, and augmented using geometric transformations and gamma corrections. SURYANI et al. [18] segmented blood vessels using a self-organizing graph artificial neural network. The proposed segmentation method is divided into three stages: preprocessing, segmentation and a performance analysis. Zhou et al. [19] proposed an end-to-end synthetic neural network to strengthen elusive vessels’ segmentation capability, containing a symmetric equilibrium generative adversarial network (SEGAN), multi-scale features refine blocks (MSFRB), and an attention mechanism (AM). Fu et al. [20] regarded retinal vascular segmentation as a boundary detection task, used multi-scale context information and a side output layer in the network to learn the rich hierarchical structure, and used conditional random fields to model the long-term dependence between pixels. Zhou et al. [21] proposed a discriminative feature learning scheme that learns effective features through a CNN for the dense CRF model. Pan et al. [22] proposed a method of retinal vessel segmentation based on an improved deep learning U-Net model, which solved the performance degradation problem of a residual network under extreme depth conditions.

**Figure 1:**
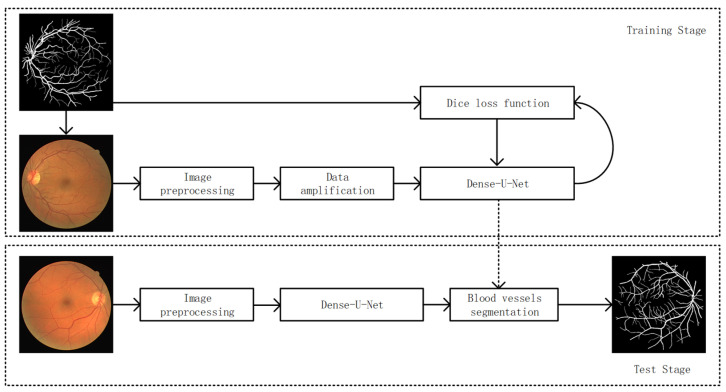
Overall flow chart.

In order to improve on the segmentation accuracy, which is not high, and small vessels segmentation incomplete problems, this paper proposes a segmentation model combining U-Net and Dense-Net. The method combines adaptive histogram equalization with contrast limitation (CLAHE), median filtering data normalization and multi-scale morphological transformation to enhance vascular-feature information. The artifact is corrected by adaptive gamma correction. The results of the pretreatment were segmented using dense-U-Net model to achieve segmentation of fine vessels. The algorithm flow is shown in Figure 1.

## 2. Principle of Retinal Image Segmentation

### 2.1. Image Preprocessing

**Figure 2:**
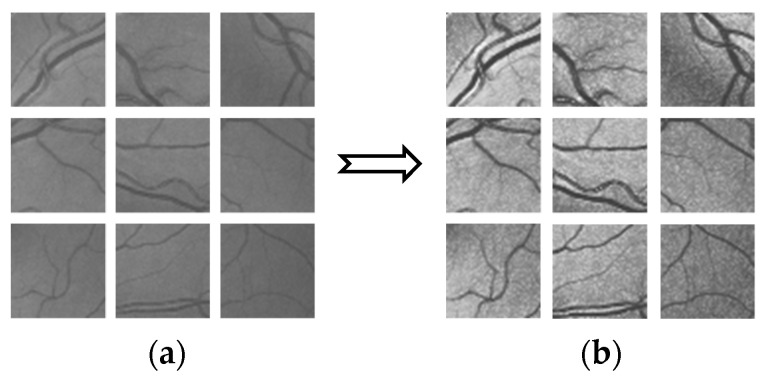
Pre-processing (**a**) original image (**b**) pre-processing results.

Due to the low background contrast and uneven illumination in the collected retinal images, the robustness of the algorithm is not high. In order to make the vascular information clearer, the original image needs to be preprocessed, for which the specific steps are as follows:

(a) The image of green channel has high contrast and low noise, so it can be used as input data.
(b) CLAHE. The contrast is improved, and noise is suppressed, so it is easier to extract vascular information.
(c) Median filtering. The lesion interference and pipeline influence were removed to better highlight the vascular characteristic information.
(d) Normalization of data. The pixel value range of the image is between (0,1), and the normalization formula is defined as follows:
  (1) $$x_{n}=\frac{x_{i}-\min\left(x\right)}{\max\left(x\right)-\min\left(x\right)}$$
  where, $x_{i}$ is the pixel value of the input image, min(x) and max(x) are the minimum pixel value and maximum pixel value in the image sample data, respectively, and $x_{n}$ is the pixel value after normalized processing.
(e) Adaptive gamma correction [23]. It is used to enhance the brightness information of the darker part of the blood vessels in the image, and can effectively retain the quality of the brighter part.
(f) Multi-scale morphological transformation [24]. By selecting four scales to control the control factors of image edge gradient information, the model is defined as:
  (2) $$f_{T}=I_{r}+k\times \sum_{i}^{n}w_{i}\left(dop_{i}-dcl_{i}\right)$$
  where, k is the image detail enhancement factor; $I_{r}$ and $f_{T}$ are, respectively the input and output images; $dop_{i}$ and $dcl_{i}$ are the features of bright detail and dark detail, respectively. The pretreatment results are shown in Figure 2.

### 2.2. Data Amplification

**Figure 3:**
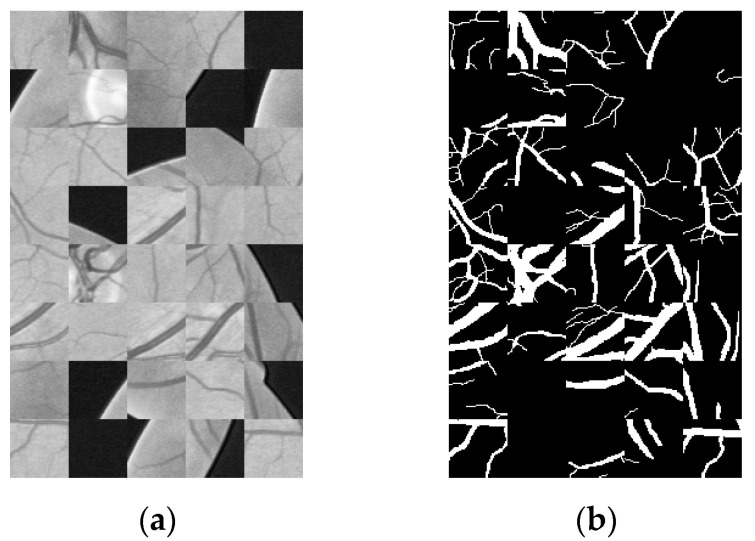
Example of local section (**a**) sample of local section (**b**) sample result of local section.

In the process of retinal vessel segmentation, a convolutional neural network can easily fall into over-fitting [25]. Data amplification is used to increase the training set and improve the generalization ability of the network model. Deep learning requires a large amount of data to fit model parameters. For the DRIVE data set, with only 20 fundus images, random slice is adopted to expand the data. According to the size of the original fundus images, the size of images extracted from the database is set to 48 × 48. Each patch with a size of 48 × 48 was obtained by randomly selecting its center within the whole image, and a total of 190,000 image blocks were extracted from the training set. Figure 3 shows an example of a partial slice.

### 2.3. Dense-U-Net Model

Convolutional neural networks can learn high-level features from low-level features, and then remove low-level features. The low reuse rate of the features does not improve the learning ability of the network effectively, and it is more meaningful to improve the utilization rate of features than to increase the network depth. In order to improve the utilization of features, a dense module is introduced, and each layer of the dense block is directly connected to all the layers before it. Dense-net uses dense blocks to improve classification performance. Dense-Net was extended to complete the convolutional network for semantic segmentation and applied to scene segmentation. However, the retinal blood vessels are small so they can be of a width of multiple pixels or even single pixels. The features of retinal vessels can be effectively learned by using dense blocks, and the segmentation accuracy of retinal vessels by U-Net based on dense blocks is higher than that of U-Net.

**Figure 4:**
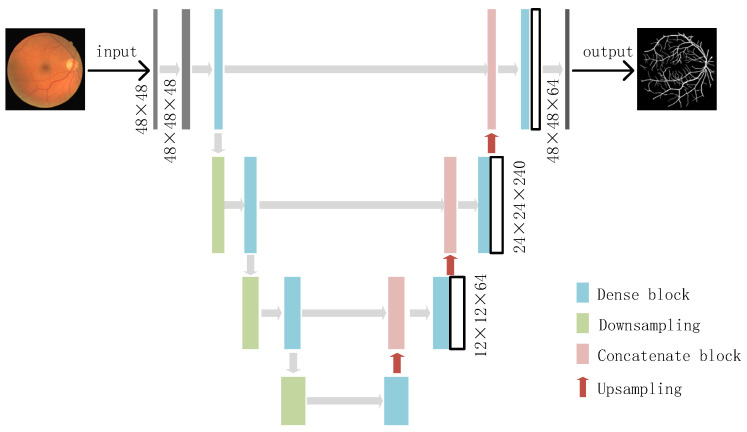
Dense-U-Net model.

Therefore, a Dense U-Net is proposed as the retinal vessel segmentation framework. A Dense U-Net is adopted as the training network, as shown in Figure 4. Image blocks were randomly extracted as training data with a resolution of 48 × 48. The model output is the prediction result, and represents the result of vascular segmentation. A dense network consists of a contraction path and an expansion path: dense blocks, transition layers and connection layers.

#### 2.3.1. Dense Block

**Figure 5:**
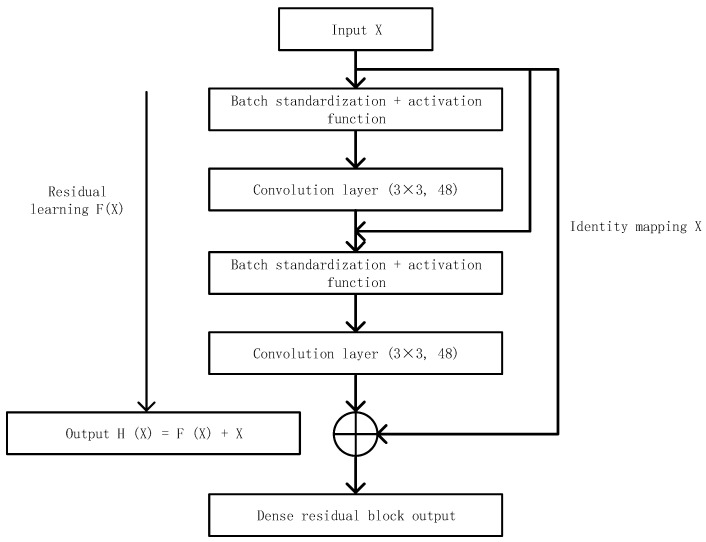
Dense residual block structure adopted in this paper.

The Dense-Net [26] design was inspired by residual networks. The similarity with residual networks is that the input of each layer is related to the preceding layer. The main difference is that Res-Net, for each layer, characterizes its input as the output of the previous finite layer. For each layer, Dense-Net’s input is characterized by the output of all the previous layers. Additionally, the output characteristics of each layer serve as the input for all subsequent layers. When the output of the layer L is $x_{L}$, it is defined as:

(3) $$x_{L}=H_{L}\left(\left[x_{0},x_{1},\cdots ,x_{L-1}\right]\right)$$

where, $\left[x_{0},x_{1},\ldots ,x_{L-1}\right]$ represents the combination of characteristic layers of layer 0,1,⋯,L−1 output; $H_{L}$ is defined as a composite function consisting of three modules: batch standardization (BN), linear correction unit (RELU) and a 3 × 3 convolution layer (CONV). The dense module is shown in Figure 5, which has L layer and generates k feature maps for each layer through a conversion function. k is the network growth rate. Assume that the number of channels of the input layer feature mapping is k_0_, then the number of channels of the output layer feature mapping is k_0_ + k × (*L* − 1).

#### 2.3.2. Loss Function

The statistical results show that only 10% of the pixels in fundus images are retinal blood vessels. The ratio of vascular and non-vascular pixels is highly unbalanced [27]. The learning process can fall into local minima of the loss function, and vascular pixels are often lost or only partially recognized.

XIE et al. [28] proposed a loss function based on class-equilibrium cross entropy. However, the loss value is affected by the weight coefficient. This method adopts a new loss function based on the Dice coefficient [29], which ranges from 0 to 1. The Dice coefficient can be defined by Equation (4):

(4) $$D=\frac{2\sum_{i}^{N}p_{i}g_{i}}{\sum_{i}^{N}p_{i}^{2}+\sum_{i}^{N}g_{i}^{2}}$$

where, *N* is the number of label pixels, *p_i_* and *g_i_* are the predicted result and ground truth, respectively, and the formula can be differentiated to generate the gradients as follows:

(5) $$\frac{\partial D}{\partial p_{j}}=2\left[\frac{g_{j}\left(\sum_{i}^{N}p_{i}^{2}+\sum_{i}^{N}g_{i}^{2}\right)-2p_{j}\left(\sum_{i}^{N}p_{i}g_{i}\right)}{{\left(\sum_{i}^{N}p_{i}^{2}+\sum_{i}^{N}g_{i}^{2}\right)}^{2}}\right]$$

## 3. Experiment

The simulation platform used in this paper is Python, based on the Tensorflow keras framework. The computer is configured with Intel(R) Core(TM) I5-7500 CPU @ 3.40 GHz, and uses a 64-bit Windows 10 operating system.

### 3.1. Experimental Data Set

DRIVE [30] includes a total of 40 fundus images, with a resolution of 565 × 584, including 20 in the training set and 20 in the test set. Each image has a gold standard with special masking, which is a commonly used database for measuring the performance of retinal vascular segmentation methods.

### 3.2. Evaluation Indicators

In order to analyze the performance of the segmentation results of the proposed algorithm, evaluation indexes such as specificity (Sp), sensitivity (Sn), accuracy (Acc), Positive Predictive Value (PPV) and AUC were used to analyze the algorithm performance, which were defined as follows:

(6) $$\mathrm{Acc}=\frac{\mathrm{TP}+\mathrm{TN}}{\mathrm{TP}+\mathrm{FP}+\mathrm{TN}+\mathrm{FN}}$$

(7) $$\mathrm{Sp}=\frac{\mathrm{TN}}{\mathrm{FP}+\mathrm{TN}}$$

(8) $$\mathrm{Sn}=\frac{\mathrm{TP}}{\mathrm{TP}+\mathrm{FN}}$$

(9) $$\mathrm{PPV}=\frac{\mathrm{TP}}{\mathrm{TP}+\mathrm{FP}}$$

where TP, TN, FP and FN are true positive, true negative, false positive and false negative, respectively. AUC represents the area under the ROC curve, ranging from 0 to 1. The greater the value of each evaluation index, the better the algorithm performance is.

### 3.3. Experimental Results and Analysis

**Figure 6:**
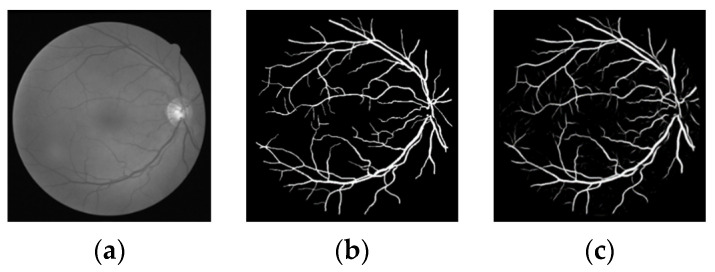
First group of segmentation results (**a**) original image (**b**) gold standard image (**c**) segmentation results of this paper.

**Figure 7:**
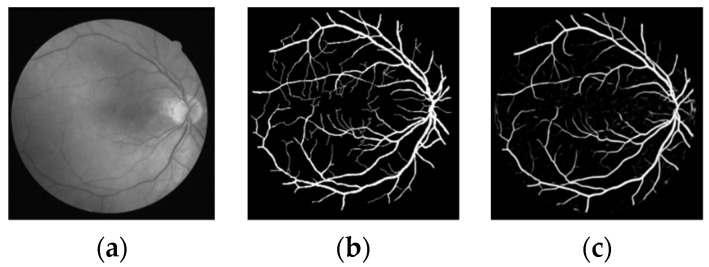
Second group of segmentation results (**a**) original image (**b**) gold standard image (**c**) segmentation results of this paper.

**Figure 8:**
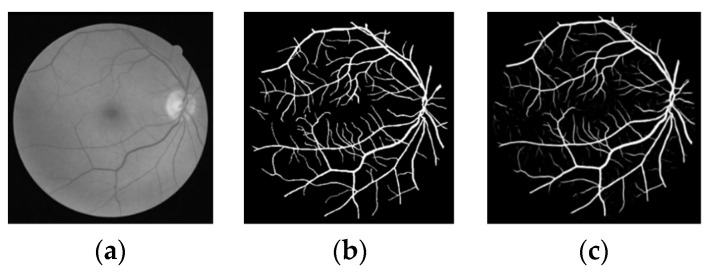
Third group of segmentation results (**a**) original image (**b**) gold standard image (**c**) segmentation results of this paper.

**Figure 9:**
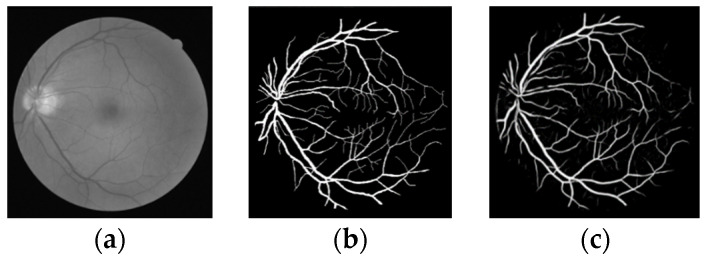
Fourth group of segmentation results (**a**) original image (**b**) gold standard image (**c**) segmentation results of this paper.

The effectiveness of this method is verified through public data set DRIVE, and the segmentation results of the Dice loss function are shown in Figure 6, Figure 7, Figure 8 and Figure 9.

As can be seen from the segmentation results in Figure 6, most of the blood vessels of the retinal fundus are accurately segmented, and some small blood vessels marked in the manual segmentation are also accurately segmented, indicating that the method in this paper has high applicability to blood vessel segmentation.

In Figure 7, there are many small blood vessels at the end of retinal blood vessels in the fundus, but in the results, only the main blood vessels and the wider blood vessels are segmented, while the small blood vessels at the end are not segmented, due to the low contrast and noise of the blood vessels. The subsequently improved methods will be studied further.

The segmentation result image in Figure 8 was compared with manual segmentation, and the unmarked blood vessels were accurately segmented at the top of the blood vessels, indicating that the method presented in this paper has high segmentation accuracy in retinal blood vessel segmentation.

In Figure 9, the segmentation is relatively accurate and microscopic blood vessels are visible, which is helpful for the diagnosis of the disease, indicating the effectiveness of the algorithm.

**Figure 10:**
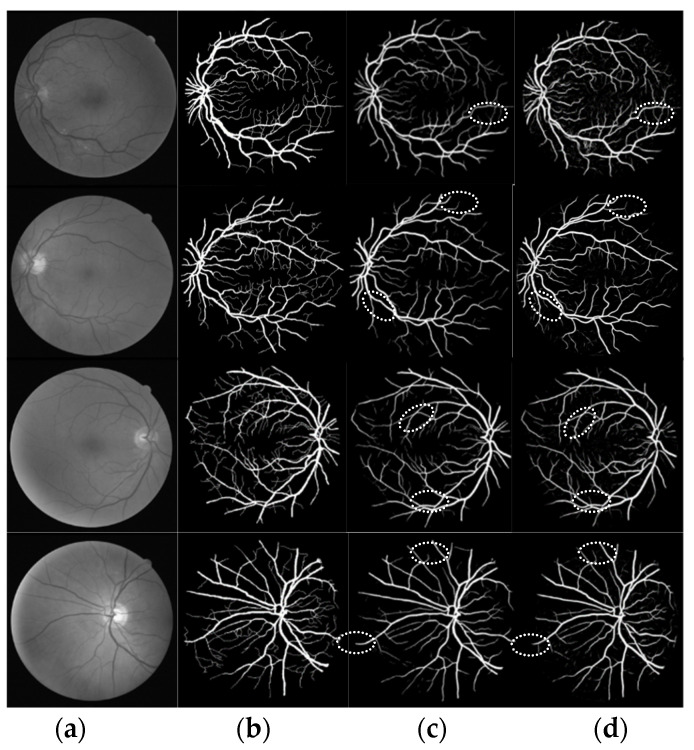
Comparison of segmentation results (**a**) original image (**b**) gold standard image (**c**) literature [22]. 2019 Pan, X. (**d**) algorithm of this paper.

To prove the feasibility of the proposed method, these results were compared with manual segmentation and segmentation in the literature. As shown in Figure 10, four images were randomly selected from the test set. Images labelled as (a) are the original image, (b) are the gold standard image, (c) are the segmentation result in [22], and (d) are the segmentation result of the algorithm in this paper. The white ellipse region is the result of fine segmentation.

As can be seen from the figure, the proposed algorithm can achieve a good segmentation effect for both normal retinal vessels and pathological retinal vessels, more micro-vessels can be segmented, and more detailed information can be segmented to better recover the vascular results of retinal images.

**Figure 11:**
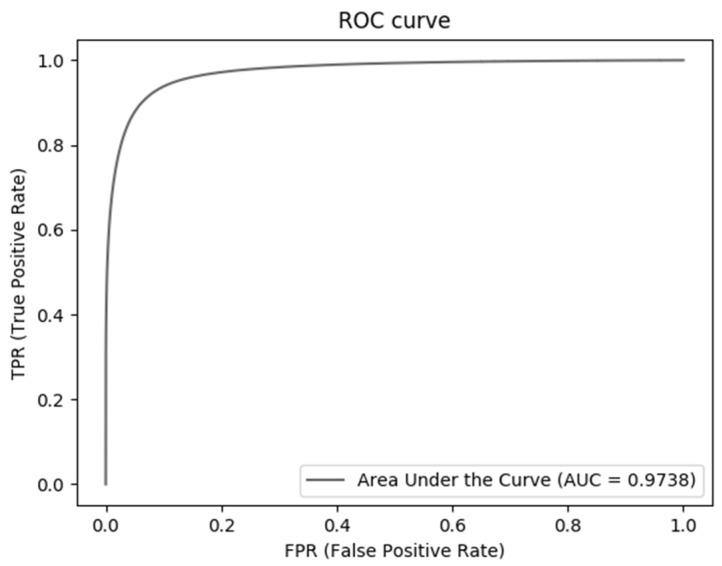
ROC curve of DRIVE data set.

In order to illustrate the advantages of the algorithm in this paper more intuitively, the ROC curve shown in Figure 11 is provided. It can be seen from the ROC curve that, in general, the performance of the proposed algorithm is superior, and the error of vessel segmentation is small.

In addition, to verify the feasibility of the proposed method, the sensitivity, specificity and accuracy of the proposed method were compared with other methods. The results are shown in Table 1. The results indicate that the accuracy, specificity and positive predictive value of the existing algorithms for vessel segmentation on the DRIVE dataset are lower than those of the algorithm in this paper, indicating that the algorithm in this paper has a better comprehensive segmentation performance on this dataset.

## 4. Conclusions

In order to solve problems of low segmentation accuracy and the incomplete segmentation of small vessels, this paper proposes a network based on the combination of U-Net and Dense-Net. Randomly extracted image blocks were used as training data, Dense-U-Net was used as training network model, random gradient descent was used to optimize Dice loss function, and random transformation was used to expand training data and improve the generalization ability. The method was applied to a public data set DRIVE to complete retinal vessel segmentation. Sn, Sp, Acc, PPV and AUC were used as evaluation indexes to verify the effectiveness of the method. The results show that the method is feasible and competitive in these evaluation indexes.

## Figures and Tables

**Table 1:** Comparison of performance data between different literature methods.

Methods	PPV	Sp	Sn	Acc	AUC
Wang [31]	-	0.9736	0.7986	0.9511	0.9740
Chen [32]	-	0.9735	0.7426	0.9453	0.9516
Strisciuglio [33]	-	0.9724	0.7731	0.9467	0.9588
Guo [34]	0.8335	0.9848	0.7891	0.9674	0.9836
Alom [35]	-	0.9813	0.7792	0.9556	0.9784
Our proposed	0.8946	0.9896	0.7931	0.9698	0.9738

## Data Availability

All data generated from this study are included in this published article. Raw data are available from the corresponding author on reasonable request.
